# Participants with mildly-disabling chronic neck pain perform differently during explicit compared to implicit motor learning of a reaching task

**DOI:** 10.1371/journal.pone.0266508

**Published:** 2022-04-07

**Authors:** Michael R. Brown, Kirkwood E. Personius, Jeanne Langan

**Affiliations:** Rehabilitation Science Department, School of Public Health and Health Professions, University at Buffalo SUNY, Buffalo, New York, United States of America; University of Ontario Institute of Technology, CANADA

## Abstract

Chronic musculoskeletal (CMSK) pain associated with musculoskeletal disorders like low back pain or neck pain are the leading causes of disability. While CMSK pain has the potential to negatively influence motor learning, there is limited research to understand the impact of CMSK on motor learning. In order to examine differences in motor learning between individuals with and without CMSK we modified a serial reaction time task to assess motor learning of a repetitive reaching task. The paradigm was used to assess both explicit and implicit motor learning. In a cross-sectional study design, seventeen participants with chronic neck pain (CNP) (5 males) and 21 controls (8 males) were recruited. In addition, physical, cognitive, sensorimotor, disability and pain assessments were used to examine differences between individuals with and without CNP. All participants with CNP were categorized as having mild disability. There was no difference in cognitive assessments and minimal differences in physical measures between groups. Examining motor learning, groups with and without CNP demonstrated similar outcomes in both explicit and implicit motor learning. There was one notable performance difference between groups in the reaching task, the group with CNP demonstrated slower reaching movements outward and inward during blocks without explicit information. This may suggest a cautious approach to movement with reduced explicit information. Findings from this study provide insight on motor learning in individuals with mildly-disabling CNP, further research is necessary to examine how instruction can impact peak performance in people with CMSK pain.

## Introduction

Chronic musculoskeletal (CMSK) pain associated with musculoskeletal disorders like low back pain or neck pain are the leading causes of disability among middle-aged adults [1–3]. Individuals with CMSK pain are more likely to experience limitations in functional movements which contribute to disability [4, 5]. In light of the opioid epidemic, the rising cost of health care associated with managing CMSK disorders, and the predicted rise in the prevalence of musculoskeletal pain-related disability [6], there is an emerging need to identify novel interventions that are safe and effective for reducing the burden of CMSK.

Physical rehabilitation offers a safe and cost-effective alternative to opioids or surgery for managing the pain, functional limitations and disability associated with CMSK pain [7, 8]. Conservative interventions such as exercise or manual therapy can be beneficial for reducing pain and improving function in individuals with CMSK pain [9–13]. However, the magnitude of the benefits for pain management, are smaller than desired and commonly short-term [14]. More recently, using motor skill training, which incorporates learning or relearning functional tasks to improve pain and reduce disability in a group of patients with chronic low back pain led to long-term improvements [15–17]. Integrating motor learning principles into rehabilitation programs could lead to better outcomes, since individuals with CMSK pain may need to acquire/re-acquire movements, motor skills and/or behaviors and then retain for daily use following rehabilitation [18, 19].

Acquisition of a motor skill (i.e. motor learning) develops from repeated practice of a specific motor task over a period of time. It is important to differentiate between actual learning and transient performance changes of the task [20]. Learned motor skills have greater stability and are less likely to degrade over time [21].

There are different forms of motor learning. Explicit motor learning refers to the learning of a motor skill via direct knowledge of how to perform the task, and relies heavily on working memory [22, 23]. In studies examining explicit motor learning, participants are provided with specific instructions (verbal, visual, or a combination of both) on how to perform the task. Implicit motor learning, on the other hand, refers to the process of acquiring a motor skill without the express knowledge of how the skill is acquired [22–24]. In studies examining implicit motor learning, participants are given information about the end goal of the task. In either explicit or implicit motor learning, learning is demonstrated when performance of the motor skill improves and is stable with disruption to practice of the skill due to a secondary activity or time. Many studies use a time-based variable (e.g. reaction time, movement time) as the main outcome measure [25].

To date, only a handful of studies have directly investigated the effect of chronic pain on explicit or implicit motor learning of simple motor tasks. For example, Vallence and colleagues [26] found impaired motor learning of a finger abduction task in a group of patients with chronic tension type headache. Parker and colleagues [27] reported motor learning of a finger task remained intact in a group of participants with painful hand arthritis. Another study comparing motor learning in a group of participants with and without sub-clinical neck pain also found similar performance on the initial motor learning of a tracing task, but performance of the skill 24-hours later revealed the control group further improved their performance without additional practice while the individuals with subclinical neck pain did not further improve [28]. The conflicting results suggests that the presence of chronic pain does not uniformly impact motor learning ability. Instead, these findings invite further investigation on how characteristics of chronic pain or the type of motor learning may influence the ability to learn the motor task.

While the presence of pain following an acute injury usually signifies tissue damage, in CMSK disorders there is less of an association between the experience of pain and the health of the tissues located at the site of the initial injury. There is the potential for maladaptive changes within the central nervous system which can lead to hypersensitivity to normal and sub-threshold sensory stimuli [29]. Similarly, cognitive impairments such as impaired attention and working memory have been reported to occur in some populations with CMSK pain [30–34]. It is possible that these changes, either one in isolation or in combination, could impair explicit or implicit motor learning of a motor skill.

The purpose of this study is to examine explicit and implicit motor learning during a reaching task for a group of participants with and without chronic neck pain (CNP). The first aim is to comprehensively characterize the participants by examining pain interference and disability, as well as physical, sensorimotor and cognitive performance. The second aim is to examine explicit and implicit motor learning performance using a repetitive reaching task.

## Methods

A cross-sectional study design was used to investigate motor learning in the presence of CMSK pain, and more specifically CNP. Participants between the ages of 18 and 35 with self-reported chronic neck pain and age similar controls with self-reported absence of pain were recruited from the community. Inclusion criteria for both groups included: participants had to be right-hand dominant, free of any acute (< three months) musculoskeletal injury to their neck or upper extremity, normal or corrected vision, no history of a neurological disease, and be able to tolerate a combination of sitting and standing for two hours. Inclusion criteria for participants with self-reported chronic pain included: current episode of neck pain greater than three months and a calculated score at or above 4% on the Neck Disability Index (NDI). Calculated scores for the NDI range from 0 to 100%, with higher scores indicating a higher degree of self-reported disability [35]. Inclusion criteria for the control participants: self-report absence of neck pain and a calculated score below 4% out of 100% on the NDI; which indicates no disability and is below the cut-off value for neck pain associated with disability [35, 36]. Participants were excluded from the study if they reported a history of surgery to their cervical spine or upper extremities within the last year. The protocol was approved by the University at Buffalo’s Institutional Review Board prior to enrolling participants in the study. Written informed consent was obtained before data collection began.

### Assessment of participant characteristics

A series of self-report, physical and cognitive measures were collected. Participants completed a demographic questionnaire modeled after the minimal data set developed by the National Institute of Health (NIH) Task Force on Chronic Low Back Pain [37] and the NDI, which is a self-reported outcome measure used to evaluate the perceived disability of patients with neck pain [35].

Clinical measures included: 1) Active range of motion (in degrees) of the cervical spine was measured using a goniometer (JAMAR EZ-Read 12.5 inch) [38]. 2) Touch localization was performed using the eraser tip of a pencil to touch a grid of six equally-spaced targets over the participant’s neck and both hands [39]. One trial of 24 touches (4 touches per target) was conducted over each area. Targets were randomly selected and different for each region. The correct number of responses was recorded 3) Pressure pain threshold testing was performed over the right and left upper trapezius muscles and right and left tibialis anterior muscles using a commercially available pressure algometer (Wagner FDX 50, Wagner Instruments, Greenwich, CT) following the protocol reported by Walton and colleagues [40, 41]. Digital cognitive assessments developed by Cogstate (Cogstate Ltd., Melbourne VIC, Australia) included: attention/reaction time, verbal working memory, and working memory.

1. The Card Detection task, an assessment of attention/reaction time, measures how quickly the participant reacts to a card turning face up on the computer screen. While sitting, participants pressed a button on the keyboard as quickly and accurately as they could the moment the card turned face up. Reaction time was recorded in milliseconds.
2. For the Grocery List task, an assessment of verbal working memory, participants were read a list of 12 common items. After hearing the list, participants were asked to repeat what they remembered. The amount of time needed to repeat what they heard and the number of correct responses were recorded. Participants completed three trials consecutively.
3. The 2-back test, an assessment of working memory, participants watched the computer screen as cards turned face up. Using the keyboard, the participant identified whether or not the card that turned face up matched the card displayed two cards ago. Reaction time in milliseconds and the number of correct responses were recorded.

### Motor learning task and setup

**Fig 1:**
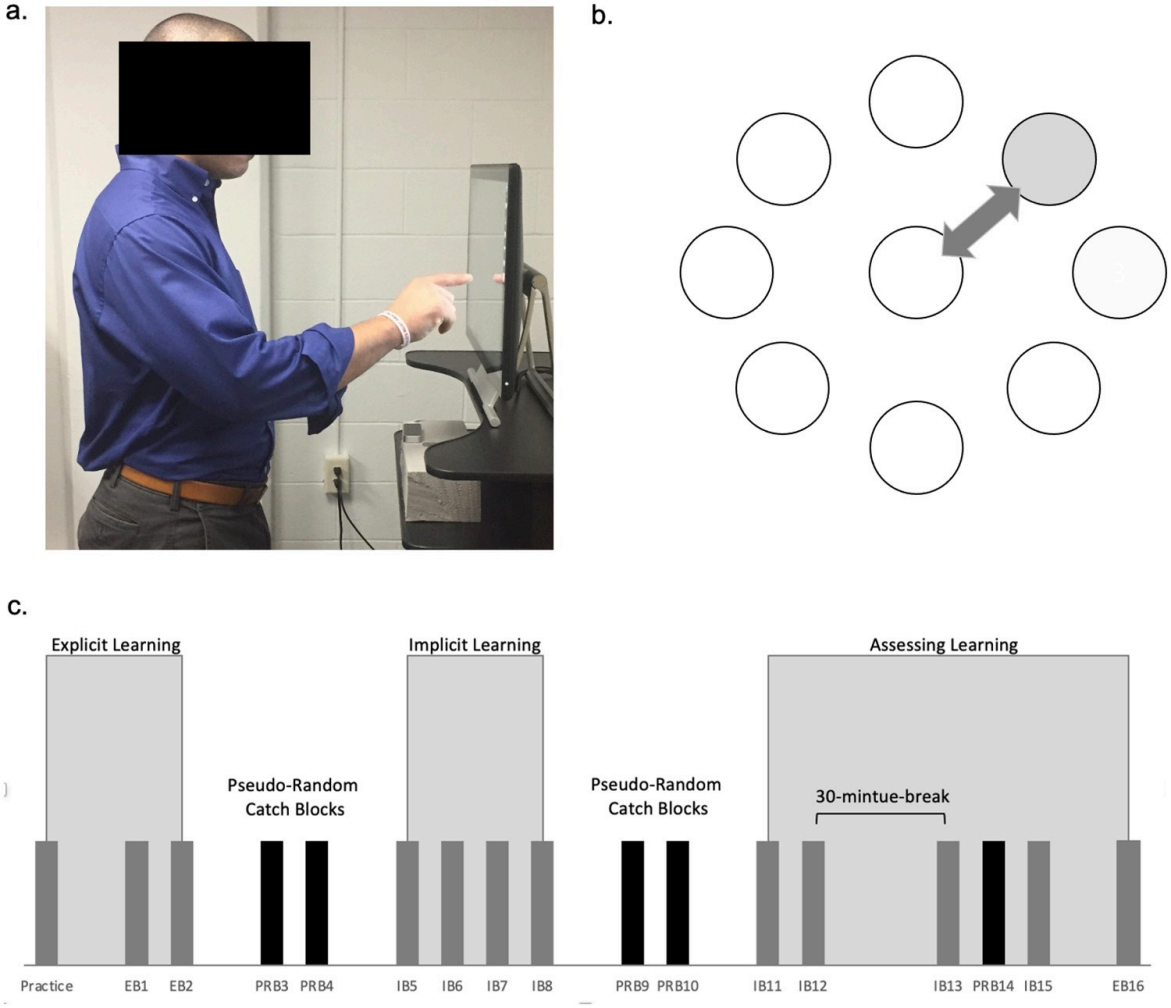
Motor learning set up and paradigm. (a) Position of participant during serial reaching task. (b) Illustration of arrangement of targets. The peripheral target (grey circle) would illuminate red indicating to the participant to reach to that target. After each reach to a peripheral target, the center target would turn red (empty circle) indicating to return to the center target. (c) Illustration of blocks of reaching movements in paradigm EB = explicit block, IB = implicit block, PRB = pseudo-random block.

The motor learning paradigm, modified from previous studies [42–44], consisted of a repetitive reaching task to targets. Participants played a custom-designed computer game developed to assess explicit and implicit motor learning in individuals with CNP. A Dell desktop, 21.5 inch touch screen monitor (Dell Technologies Inc., Round Rock, Tx) was used to display the targets and register the participants’ touch during the task. A Leap motion infrared sensor (Leap Motion, https://leapmotion.com) was placed beneath the touch screen monitor to capture participants’ hand motion. A laptop computer (Dell Technologies Inc., Round Rock, Tx) was used to run the game, record and store the data. The sampling time was 0.0165 seconds [45]. The touch screen was situated on an adjustable height table and sit-to-stand desk riser. Participants were instructed to stand behind a piece of tape on the floor while the table height was adjusted so that shoulder elevation remained below 90 degrees of flexion during reaching outward movements (Fig 1a).

Eight peripheral targets arranged in a circle surrounding a central target were displayed on a digitized touch screen (Fig 1b). The center of each peripheral target was oriented 45 degrees from the previous target. While participants did not see numbers on the targets in the custom-designed computer game, for the sake of clarity in describing the task the targets are referred to by number starting at the top of the screen: target 1 = 0^0^, target 2 = 45^0^, target 3 = 90^0^, target 4 = 135^0^, target 5 = 180^0^, target 6 = 225^0^, target 7 = 270^0^, target 8 = 315^0^. The central target is numbered 0. Each peripheral target was 11 cm from the center of the central target. All targets were 2.5 cm in diameter. Targets changed color from yellow to red signifying to the participant it was the intended target. Once the red target was touched, the color returned to yellow and the next intended target turned red. Participants made both outward and inward movements alternating between the central target turning red and peripheral targets turning red.

### Explicit motor learning

During the assessment of explicit motor learning, participants were provided with information on how to complete the sequence of reaching movements. Participants received information that the peripheral targets would illuminate in a clockwise direction. Using target numbers for clarity, the repeating pattern would be: 0, 1, 0, 2, 0, 3, 0, 4, 0, 5, 0, 6, 0, 7, 0, 8. Participants were instructed to move in a clockwise direction, returning to the central target after reaching out to each peripheral target in the outer ring. A visual demonstration followed the verbal instructions so participants could visualize the task of reaching out and in from the center target to the peripheral target and back. After the explanation of the task, participants were given an opportunity to practice touching the targets, reaching out and in, two times around the circle of targets in order to famiiarize themselves with the task.

### Implicit motor learning

To examine implicit motor learning a repeating sequence of targets was presented to participants without their knowledge. Participants started at the center target and reached for the target in the outer circle followed by reaching back to the center target. The repeating sequence of targets during the implicit motor learning condtion was: 0, 8, 0, 2, 0, 7, 0, 5, 0, 3, 0, 1, 0, 4, 0, 6. Since it is likely that participants would quickly anticipate the inward movement to the center target, movement outward to the peripheral targets and movement inward to the center target were examined independently.

### Overview of motor learning paradigm

Each motor learning condition was grouped into a series of blocks (Fig 1c) consisting of 32 movements outward and 32 movements inward, separated by 30 second breaks between blocks to avoid fatigue.

Participants were instructed to move as quickly and accurately as possible. They received a three second audiovisual count down and then quickly reached to the red central target, then to the red target in the outer ring; then back to the red central target. They were required to touch anywhere inside the red target with their index finger; touches outside the intended target kept the participant from moving on to the next target in the sequence.

Participants completed a total of two consecutive explicit motor learning blocks (EB1 and EB2). Two pseudo-random catch blocks immediately followed (PRB3 and PRB4). During a pseudo-random catch block, targets in the peripheral circle of targets appeared in random order. In a pseudo-random block, participants completed the same movements outward to the peripheral target and inward to the center target as in the learning blocks, but the order of presentation of the red peripheral target was not learnable. Pseudo-random catch blocks served as comparisons for the explicit motor learning blocks. If the participants explicitly learned how to perform the motor task, it was expected that the time to complete the explicit motor learning block would be less than the pseudo-random catch blocks.

A total of four implicit motor learning blocks (IB5, IB6, IB7 and IB8), were completed. Two pseudo-random catch blocks (PRB9 and PRB10) followed. Again, it was anticipated that participants’ time to complete the pseudo-random catch block would increase compared to performance at the end of implict motor learning, IB8. To verify the sequence was learned, participants performed two more blocks with the implicit motor learning sequence. It was predicted that performance times would be less than the pseudo random catch block if the participants learned the sequence of targets. Finally, an assessement after a brief passage of time was conducted to determine if performance of the task deteriorated over time. Participants stopped the reaching task for 30 minutes. During the break they completed the digital cognitive assessments. Following the 30-minute break participants performed the implicit motor learning sequence again. If they acquired the skill of performing the repeating sequence, they would perform similarly to the last implicit motor learning block prior to the break.

To confirm that participants had implicitly motor learned the sequence, without awareness of the sequence of targets, participants were asked at the end of the study if they noticed a repeating sequence of targets at any point during the study. Participants were handed a paper-based test containing a blank circle of peripheral targets surrounding a central target. Participants were instructed to indicate the order in which they felt the peripheral targets were presented by labeling the circles in the outer ring from one to eight, with one representing the first target in the sequence and eight representing the last target.

### Data analysis

Data was analyzed using SPSS (IBM Corp. Released 2017. IBM Statistics for Macintosh, Version 25.0. Armonk, NY: IBM Corp.). Descriptive statistics were calculated for age, body mass index, pain intensity, scores on the NDI, as well as results from the cognitive tests and physical measures. A two-tailed independent T-test was used to examine between-group differences for select demographic variables (e.g. age), physical measures (e.g. range of motion), and cognitive measures.

Primary variables of interest for the two motor learning conditions included the cumulative time spent reaching outward toward the peripheral targets and the cumulative time spent reaching inward to the center target during an entire block. Table 1 outlines the planned comparisons used to establish explicit and implicit motor learning. Secondary variables of interest included the total hand path distance reaching outward to the peripheral targets, the total hand path distance reaching inward to the center target, and the total number of errors committed during a block.

**Table 1:** Planned comparisons to establish explicit and implicit motor learning.

Measures	Explicit blocks	Implicit blocks
Changes in performance over continuous motor learning blocks	Average (avg.) time reaching outward in EB2 –avg. time reaching outward in EB1	Avg. time reaching outward in IB8 –avg. time reaching outward in IB5
Comparison of pseudo-random catch block to motor learning block to examine acquisition of repeating sequence, motor learning	Avg. time reaching outward in PRB3 –avg. time reaching outward in EB2	Avg. time reaching outward in PRB9 –avg. time reaching outward in IB8
Comparison of motor learning blocks across time to examine retention of repeating sequence, motor retention	Avg. time reaching outward in EB16 –avg. time reaching outward in EB2	Avg. time reaching outward in IB11 –avg. time reaching outward in IB8
		Avg. time reaching outward in IB13 –avg. time reaching outward in IB12

The planned analysis of the primary and secondary variables included parametric statistics; however, both the primary and secondary variables of interest were tested for normality and found to be non-normally distributed. To avoid overworking the data, a conservative approach to analyzing the results was adopted [46]. Accordingly, a Sign test was used to examine within-group differences in performance for select blocks within the explicit and implicit motor learning conditions. A Mann-Whitney U test was used to analyze performance differences between the group distributions for the same blocks within the explicit and implicit motor learning conditions.

In order to preserve the integrity of the results for implicit learning, individuals who correctly identified the repeating sequence of targets after practice were removed from the implicit motor learning analysis. Knowledge of the sequence could have influenced their performance of the reaching task.

## Results

A total of 44 participants between the ages of 18 and 35 consented to participation in the study. The number of participants in the final analyses was 38 due to exclusion of six participants from the control group. Five control participants who reported no history of neck pain did not meet the inclusion criteria of a score below four percent on the NDI. Their NDI scores ranged from four to ten percent. One participant was excluded due to experimental error during data collection. The final analysis contained 21 participants (8 males) in the control group and 17 participants (5 males) in the CNP group. A two-tailed Independent T-test indicated there were no significant differences between the groups for age or BMI.

### CNP participant characteristics

According to the results from the NDI, participants with CNP presented with a mild level of disability associated with their neck pain [35]. Their average score on the NDI was 20% with a range of 6–40%. Participant’s history of CNP ranged from three months to greater than five years, with 59% (n = 10) reporting CNP between one and five years. Six participants with CNP (35%) experienced neck pain on a daily basis or nearly every day over the past six months. Seven participants (41%) reported pain at least half of the days over the past 6 months. The four remaining participants with CNP (24%) reported experiencing pain less than half the days over the past 6 months. The location of pain reported by the participants with CNP varied, with just over half experiencing symptoms bilaterally (n = 10, 59%). Only four participants (24%) reported experiencing upper extremity symptoms during the two weeks before testing. Participants with CNP reported occurrences of pain in areas other than their neck including, stomach pain (53%), other musculoskeletal pains (53%), widespread pain (29%), and headache (94%) in the weeks leading up to participating in the study. Nine participants with CNP (53%) also rated their quality of sleep less than good. Four individuals with CNP (24%) indicated they had reservations about how physical activity might influence their neck pain. Finally, 76% of the participants with CNP reported feeling pain at the start of testing. Table 2 lists the average pain values for the week prior to testing, the moment prior to testing, and post-testing.

**Table 2:** Descriptive statistics for self-reported measures.

*Variable*	Control		CNP		^a^ *P-value*
	*M*	*SD*	*M*	*SD*	
Age (years)	24.76	3.85	24.47	3.69	0.815
BMI (kg/m2)	25.12	4.52	24.65	4.48	0.751
NDI	0.95	1.02	20.94	9.06	<0.001
Avg. pain previous week (NPRS)	-	-	4.18	2.51	-
Pain prior to testing (NPRS)	-	-	2.47	2.04	-
Pain after testing (NPRS)	-	-	2.76	2.05	-

Note: BMI = body mass index in kg/m2, NDI = neck disability index, NPRS = numeric pain rating scale where 0 = no pain and 10 = worst imaginable pain, ms = milliseconds.

^a^ p-values were calculated using an independent t-test.

### Cognitive assessments and clinical measures

Table 3 lists the descriptive statistics for the cognitive assessments for both the control group and the group with CNP. Participants with CNP performed similarly to the control group on all three tests, between group measures did not reach statistical significance.

**Table 3:** Descriptive statistics for cognitive measures.

*Variable*	Control		CNP		^a^ *P-value*
	*M*	*SD*	*M*	*SD*	
Verbal working memory: Duration (ms)	181832	24337.16	185378.59	37274.69	0.726
Verbal working memory: Correct responses	28.67	4.18	27.65	3.30	0.417
Attention: Speed (ms)	2.45	0.05	2.46	0.04	0.426
Attention: Errors	0.81	1.33	0.82	1.24	0.974
Working memory: Speed (ms)	2.86	0.08	2.85	0.10	0.771
Working memory: Errors	2.52	1.99	3.06	2.08	0.424

Note: ms = milliseconds,

^a^ p-values were calculated using an independent t-test.

Table 4 lists the descriptive statistics for the clinical measures. Only AROM and touch localization differed statistically across groups. First, the CNP group presented with significantly less AROM into left rotation (p < 0.001); a trend of less AROM into right rotation was also noted (p = 0.084). Second, participants with CNP had more incorrect responses during touch localization of the left hand compared to the control group (p = 0.046).

**Table 4:** Descriptive statistics for clinical measures.

*Variable*	Control		CNP		^a^ *P-value*
	*M*	*SD*	*M*	*SD*	
**Cervical Spine AROM (degrees)**					
Flexion	53.76	10.14	50.65	11.54	0.382
Extension	58.43	9.85	52.59	12.97	0.123
Left Lateral Flexion	33.38	6.87	33.06	9.18	0.902
Right Lateral Flexion	32.48	5.62	33.24	8.75	0.748
Left Rotation	77.43	3.53	69.88	7.42	<0.001
Right Rotation	75.67	5.01	72.18	7.09	0.084
**Touch Localization (# correct)**					
Left hand	21.1	2.21	19.47	2.63	0.046
Right hand	21.38	2.46	19.53	3.43	0.061
Neck	21.43	1.94	20.71	1.96	0.263
**Pressure Pain Threshold (kgf)**					
Left Upper Trapezius	5.87	1.81	6.15	3.05	0.730
Right Upper Trapezius	5.75	1.72	5.99	2.79	0.748
Left Anterior Tibialis	11.81	6.16	11.32	5.16	0.795
Right Anterior Tibialis	12.94	6.56	11.67	6.40	0.552

Note: BMI = body mass index in kg/m2, NDI = neck disability index, NPRS = numeric pain rating scale where 0 = no pain and 10 = worst imaginable pain, ms = milliseconds, kgf = kilogram pounds per foot.

^a^ p-values were calculated using an independent t-test.

### Evidence of explicit motor learning

**Fig 2:**
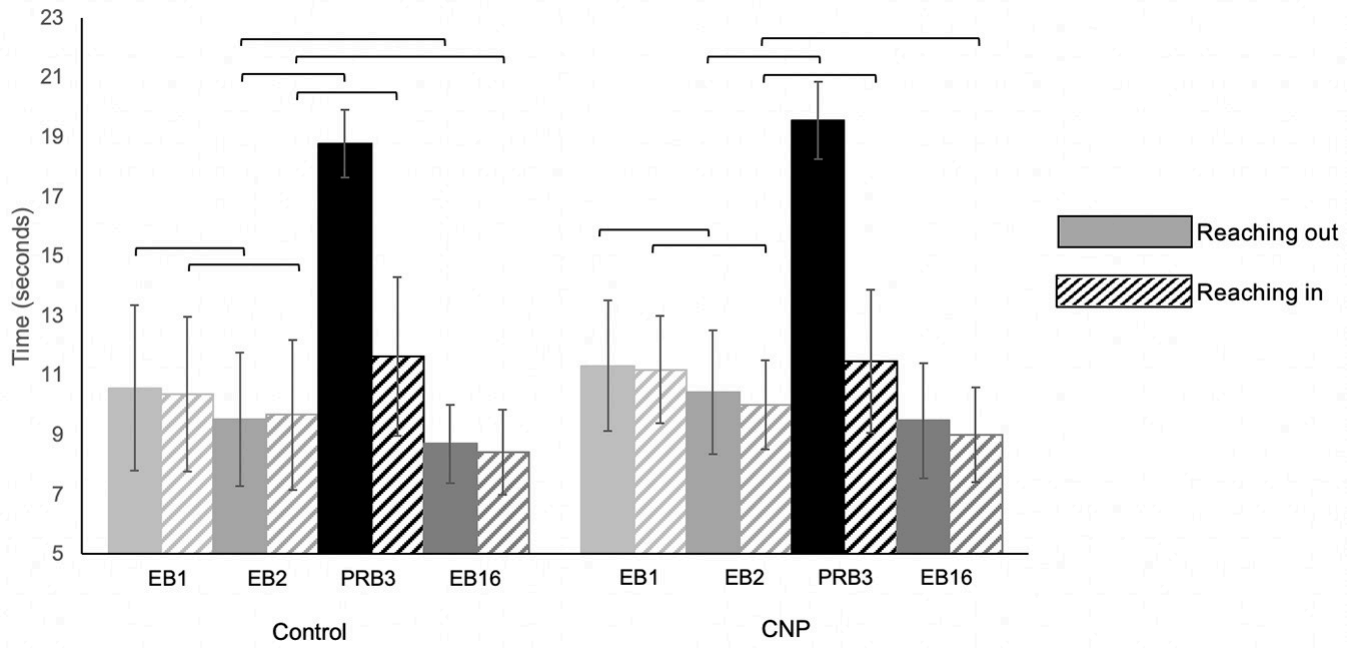
Explicit motor learning. Following practice, participants completed two blocks of outward and inward reaching to a known pattern of targets (EB1 and EB2). Bars represent the average time to complete the upper limb reaching task. Reaching outward movements are represented by solid bars, inward reaching movements by striped bars. After the two explicit motor learning blocks a pseudo-random catch block (PRB3) was introduced to assess explicit motor learning. The stability of explicit motor learning was later assessed at the end of the trial in EB16. Error bars represent standard deviations. Brackets indicate a significant within-group difference according to a Sign test, p < 0.05.

According to a Sign test, both the control group and the group with CNP demonstrated a significant within-group decrease in the amount of time spent reaching outward to the peripheral targets (p = 0.007 and p = 0.013), as well as inward to the center target (p = 0.007 and p = 0.027) during the EB2 compared to EB1 (Fig 2). Both the control and CNP group spent more time reaching outward to the peripheral targets (p < 0.001), as well as inward to the center target (p < 0.001 and p = 0.013, respectively) during pseudo-random catch block RB3 compared to EB2. No significant difference between the time spent reaching out during EB16 and EB2 was noted for the group with CNP (p = 0.332); however, they spent significantly less time reaching in (p = 0.013). The control group spent significantly less time reaching out and in during EB16 compared to EB2, (p = 0.027 and p = 0.001 respectively).

A Mann-Whitney U test, exploring performance differences between the control group and the group with CNP distribution during the three explicit motor learning blocks and the pseudo-random catch block indicated the groups did not differ significantly from each other in all four blocks, see Table 5 for results.

**Table 5:** Explicit motor learning within group and between group differences.

	**Control: Reaching outward**			**CNP: Reaching outward**			Mann-Whitney	
*Block*	*M (sec)*	*SD*	^a^ *p-value*	*M (sec)*	*SD*	^a^ *p-value*	*U*	^e^ *p-value*
EB1	10.57	2.77		11.32	2.19		129	0.091
EB2	9.54	2.24	^b^ 0.007	10.45	2.08	^b^ 0.013	132	0.172
PRB3	18.78	1.12	^c^ <0.001	19.56	1.29	^c^ <0.001	153	0.454
EB16	8.69	1.38	^d^ 0.027	9.62	1.93	^d^ 0.332	133	0.182
	**Control: Reaching inward**			**CNP: Reaching inward**			Mann-Whitney	
*Block*	*M (sec)*	*SD*	^a^ *p-value*	*M (sec)*	*SD*	^a^ *p-value*	*U*	^e^ *p-value*
EB1	10.38	2.60		11.20	1.80		119	0.081
EB2	9.68	2.52	^b^ 0.007	10.01	1.50	^b^ 0.027	141	0.271
PRB3	11.65	2.66	^c^ <0.001	11.47	2.41	^c^ 0.013	175	0.918
EB16	8.46	1.49	^d^ 0.001	9.12	1.57	^d^ 0.013	132	0.172

^a^ p-values calculated using a Sign test.

^b^ = EB2 –EB1 (comparison of performance change over explicit motor learning blocks).

^c^ = PRB3 –EB2 (comparison of pseudo-random catch block to explicit motor learning block).

^d^ = EB16 –EB2 (comparison of explicit motor learning over time).

U = Mann-Whitney U test statistic,

^e^p-values calculated using a Mann-Whitney U test.

Due to sensor error, two participants from the control groups hand path data was not included in the analysis. Neither the control group or the group with CNP demonstrated a statistically significant within group difference in the hand path distance reaching outward to the peripheral targets between EB2 compared to EB1 or EB16 compared to EB2 (p > 0.05 respectively). A significant within group difference in hand path distance reaching outward to the peripheral targets was noted between PRB3 compared to EB2 for both the control group (p < 0.001) and the group with CNP (p < 0.001). Comparisons of the hand path distance reaching inward for both the control group and the group with CNP did not reach a reliable level of significance, see S1 Table for results.

Neither group reached a statistically significant difference in the number of errors with practice of the known sequence in EB2 compared to EB1 (p > 0.05). The control group committed significantly less errors during PRB3 (p = 0.006), while the group with CNP failed to reach a reliable statistical difference (p = 1.000). No statistical difference was noted between EB16 and EB2 for both groups. Additionally, there was no statistical difference between the groups for any of the blocks according to a Mann-Whitney U test (p’s > 0.05). See S2 Table for results.

### Evidence of implicit motor learning

**Fig 3:**
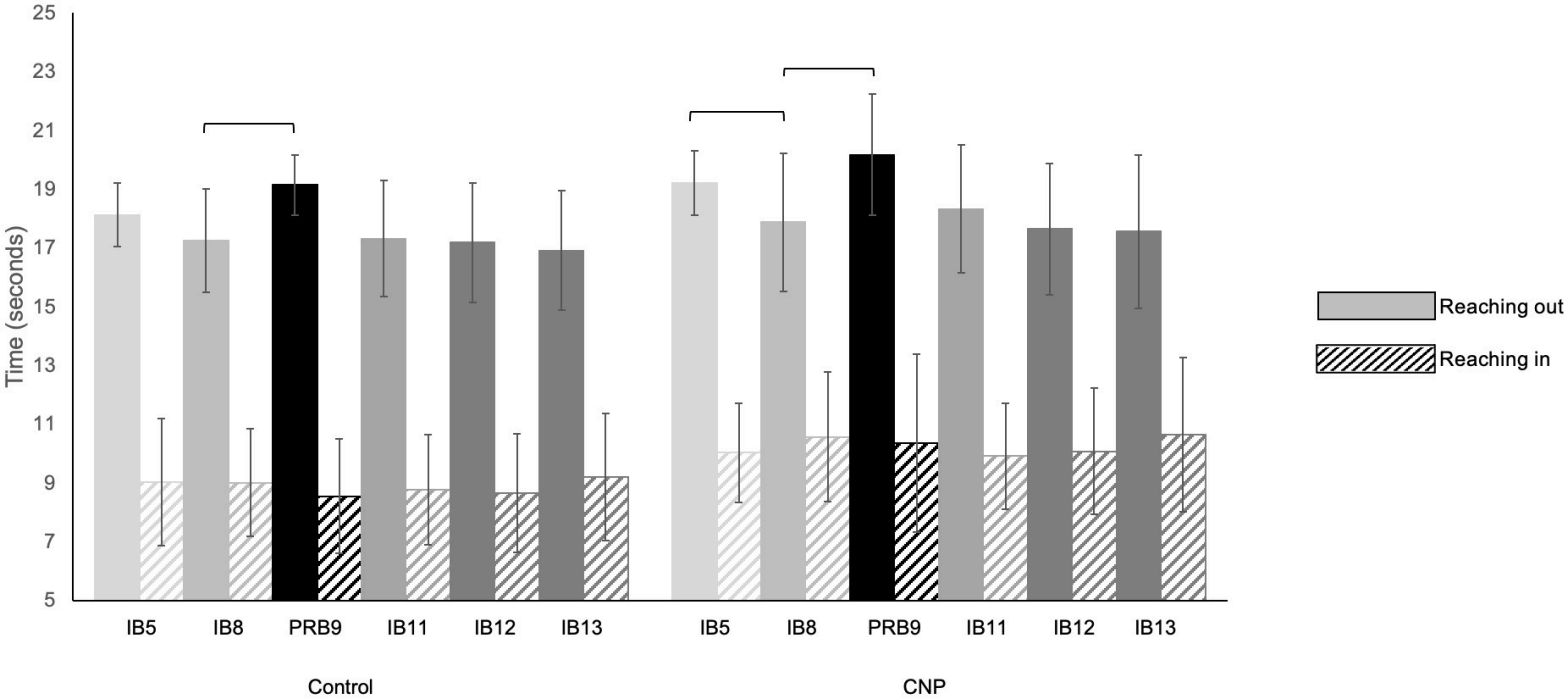
Implicit motor learning. The implicit sequence was introduced in IB5 and practiced continually through IB8. A pseudo-random catch block (PRB9) immediately followed IB8 to assess implicit motor learning. The stability of implicit motor learning was assessed in implicit block (IB11) following disruption from the pseudo-random catch block and following a 30-minute delay (IB13). Reaching outward movements are represented by solid bars, inward reaching movements by striped bars. Error bars represent standard deviations. Brackets indicate a significant within-group difference according to a Sign test, p < 0.05.

Examining within group performance, participants with CNP demonstrated improved motor performance when comparing the last implicit block, IB8 to the first implicit block, IB5 (Fig 3). Outward reaching to the peripheral target was faster by IB8 (p = 0.021). There was not a reliable difference between IB8 and IB5 outward reaching time for the control group (p = 0.064). Time spent reaching inward toward the center target remained the same for both groups during the entire implicit learning series (p > 0.05). Both the control and the group with CNP spent significantly more time reaching out to the outer circle of targets during the pseudo-random catch block (PRB9) compared to the last implicit motor learning block (IB8) (p < 0.001 and p = 0.004 respectively) (Fig 3). Time spent reaching inward toward the center target remained statistically indistinguishable for both groups (p > 0.05).

Despite being interrupted by the pseudo-random catch block no significant differences reaching outward or inward for either the control group or group with CNP were noted when comparing IB11 to IB8 (p > 0.05 respectively). Similarly, comparing the time spent reaching outward and inward during IB13 (following the 30-minute break) to IB12, both groups spent the same amount of time reaching outward to the peripheral targets or inward to the center target (p > 0.05).

Analysis of the implicit motor learning blocks with a Mann-Whitney U test revealed between group differences in performance of the implicit motor learning paradigm (Table 6). the control group spent less time reaching outward the first time groups were introduced to the repeating sequence of targets in IB5 (p = 0.008). The control group also spent less time reaching out during the first pseudo-random catch block, PRB9 compared to the group with CNP (p = 0.034). Examining the time to reach inward, the control group reached inward significantly faster than the group with CNP in IB5 (p = 0.034), IB8 (p = 0.029), PRB9 (p = 0.017), IB11 (p = 0.026), IB12 (p = 0.035).

**Table 6:** Implicit motor learning within group and between group differences.

	**Control: Reaching outward**			**CNP: Reaching outward**			Mann-Whitney	
*Block*	*M (sec)*	*SD*	^a^ *p-value*	*M*	*SD*	^a^ *p-value*	*U*	^f^ *p-value*
IB5	18.12	1.08		19.21	1.09		73	0.008
IB8	17.26	1.76	^b^ 0.064	17.87	2.35	^b^ 0.021	121	0.317
PRB9	19.15	1.02	^c^ <0.001	20.18	2.05	^c^ 0.004	88	0.034
IB11	17.32	1.98	^d^ 1.000	18.32	2.18	^d^ 1.000	115	0.230
IB12	17.18	2.03		17.64	2.24		130	0.481
IB13	16.91	2.03	^e^ 0.064	17.57	2.61	^e^ 0.359	130	0.481
	**Control: Reaching inward**			**CNP: Reaching inward**			Mann-Whitney	
*Block*	*M (sec)*	*SD*	^a^ *p-value*	*M*	*SD*	^a^ *p-value*	*U*	^f^ *p-value*
IB5	9.03	2.16		10.03	1.68		88	0.034
IB8	9.01	1.84	^b^ 1.000	10.57	2.21	^b^ 1.000	86	0.029
PRB9	8.55	1.95	^c^ 0.167	10.37	3.03	^c^ 0.454	80	0.017
IB11	8.78	1.87	^d^ 0.167	9.91	1.80	^d^ 0.210	85	0.026
IB12	8.65	2.01		10.08	2.14		89	0.035
IB13	9.21	2.17	^e^ 0.359	10.65	2.64	^e^ 0.077	101	0.095

^a^ p-values calculated using a Sign test.

^b^ = IB8 –IB5 (comparison of performance change over implicit motor learning blocks).

^c^ = PRB9 –IB8 (comparison of pseudo-random catch block to implicit motor learning block).

^d^ = IB11 –IB8 (retention of implicit motor learning following disruption).

^e^ = IB13 –IB12 (retention of implicit motor learning over time).

U = Mann-Whitney U test statistic,

^f^ p-values calculated using a Mann-Whitney U test.

In addition to removing the participants who learned the pattern of targets, two participant’s hand path data from the implicit blocks was not included in the analysis secondary to a sensor error. The control group covered significantly less hand path distance reaching outward to the peripheral targets in IB8 compared to IB5 (p = 0.013), while the group with CNP did not reach a statistically significant difference (p = 0.077). The control group covered significantly more hand path distance when the pseudo-random catch block (PRB9) was introduced compared to IB8 (p = 0.002). Again, the group with CNP did not reach a statistically significant difference in hand path distance (p = 0.077). None of the remaining comparisons reaching outward to the targets, or inward to the center target reached a statistically significant difference. See S3 Table for results.

Neither group demonstrated a significant within group difference in the total number of errors committed during a block for IB5, IB8, PRB9, IB11, IB12, and IB13. However, the group with CNP committed significantly fewer errors compared to the control group during IB8 (p = 0.036). See S4 Table for results.

## Discussion

One of the goals of this study was to better characterize the participants with CNP, with the intent of identifying characteristics that potentially could impair the acquisition of a new motor skill. Findings from this study indicate that the participants with CNP were mostly young individuals with mild disability according to the average score on the NDI [35]. There were no differences in performance on cognitive assessments and few differences in the clinical measures between the two groups. The main difference between the control group and group with CNP was the presence of mild CNP. Extrapolating the results of the study beyond individuals with mild CNP is cautioned as it is possible that higher levels of pain, or self-reported disability, or a combination of both may influence performance on the cognitive assessments, clinical measures, or the upper limb reaching task. Previous research examining changes in cognitive performance in individuals with CMSK pain [30–34], or motor learning [26–28] have inconsistently reported levels of pain. Further research is necessary to better understand if higher levels of pain influence cognitive performance and or motor learning.

The primary goal of this study was to determine if individuals with CNP acquired a motor skill similarly to that of age-matched controls. Results suggested similar performance between groups in explicit motor learning. This parallels research findings from a study investigating motor learning of a tracing task in a group of young participants with sub-clinical neck pain (age range: 20–28) [28] and another study examining finger movement in older individuals with CMSK pain as a result of arthritis (average age = 72, SD = 6) [27]. These studies suggest that the ability to explicitly motor learn in the presence of CMSK is not dependent on age. Additionally, this study and the results from Parker et al. [27] also suggest that including movement of or around the body part impacted by CMSK pain does not impact explicit motor learning. Contrary to the findings presented in this study, Vallence and colleagues [26] demonstrated that chronic pain was detrimental to motor learning in a group with chronic tension type headache compared to healthy controls. The current study enrolled participants with neck pain while Vallence et al. [26] enrolled individuals with chronic tension type headache; however, it is important to note that the majority of participants who presented with CNP in this study reported experiencing headaches in the weeks leading up to the study (CNP with headache = 94%). Vallence and colleagues [26] also did not find a correlation with headache intensity.

The difference in outcomes between studies suggests the different paradigms should be further examined. Both Vallence et al. [26] and Parker et al. [27] expressed motor learning as a function of cortical excitablity following training in a simple motor task (thumb aduction and index finger abduction respectively). Andrew et al. [28] used a specially designed tracing task to assess sensory evoked potentials. The current study chose a behavioral approach that measured the change in performance time of a complex reaching task. Additionally, the current study assessed motor learning in participants who presented with pain the day of testing. Only two of the studies [26, 27] reported testing participants experiencing pain at the time of testing; however, they did not report the intensity of pain on the day of testing. On average, participants with CNP in the current study reported a low level of pain intensity during testing which may have not impaired their ability to perform the upper limb reaching task. Examining both physiological and behavioral outcome measures of more complex motor tasks in future studies may provide further insight into motor learning with CMSK pain.

This study demonstrates that implicit motor learning is preserved in individuals with mildly-disabling, self-reported CNP. This suggests that the complex neurophysiologic process of processing sensorimotor information relevant to repetitive reaching tasks [47–49] remains intact despite the presence of persistent pain. These results align with evidence from other studies examining implicit motor learning in non-musculoskeletal conditions and older adults [50].

There were some notable differences in performance between the groups during the implicit motor learning condition that were not present during the explicit motor learning condition. Participants in the group with CNP performed the outward reaching movements significantly slower when the implicit motor task was introduced, as well as during the pseudo-random condition. Additionally, the group with CNP consistently spent significantly more time reaching inward to the center target compared to the control group. It’s possible that the group with CNP might have approached the reaching tasks without explicit information more cautiously compared to controls to prevent increases in pain levels, similar to a conditioned response to avoid pain during an unfamiliar task [51, 52]. Both the expectation and experience of pain has been shown to influence behavior [53–56]. Participants in the group with CNP could have spent more time during the reaching task secondary to gauging how the task influenced their CNP in addition to learning how to perform the task. Interestingly, performance reaching inward, the predictable movement in the sequence, was more commonly slower than the control group. This suggests that lack of explicit information for part of the task can impact performance throughout the task. Future research investigating motor learning in participants with CMSK pain should consider the cognitive-affective aspects, especially how a lack of explicit information could potentially influence performance during motor learning [57].

It was anticipated that examining changes in the number of errors and hand path distance could help identify movement strategies underlying changes in time for both explicit and implicit motor learning conditions. In the explicit motor learning blocks the outward hand path for both groups was shorter when they had knowledge of the path compared to outward movement in the pseudo-random catch block, while the number of errors did not help explain the shorter times in the explicit blocks. Examining implicit motor learning, only the control group reached statistical significance reducing their hand path distance reaching outward to the peripheral targets following practice, as well as in comparison to the pseudo-random catch blocks. This diverges from the group with CNP who did not reach a level of statistical significance (p = 0.077). This leaves the possibility that a combination of variables are influencing performance changes in the group with CNP and further research is necessary to tease out the contributing factors.

## Limitations

Results from this study should be interpreted with care since the sample population is not entirely representative of all individuals with CNP. Individuals included in this study self-identified as having chronic neck pain and were not diagnosed with having chronic neck pain. The participants with CNP had low levels of self-reported disability, low levels of pain, and lacked cognitive and sensory impairments which could have impacted their willingness to participate in the study, and thus creating a selection bias. It is also possible participants with higher levels of self-reported disability, cognitive and/or sensory processing impairments could have performed more poorly on the upper limb reaching task. While the study results provide insight into the influence of mildly-disabling neck pain on motor learning, extrapolating the findings to individuals with moderate or severely disabling neck pain is not warranted.

## Conclusions

The results from this study demonstrate that participants with mildly-disabling self-reported CNP, without cognitive impairments and few clinical impairments, were able to explicitly and implicitly learn a repetitive upper limb reaching task similar to controls without a history of neck pain. Unexpectedly, motor performance during implicit but not explicit learning of the repetitive upper limb reaching task showed participants with CNP had longer movement times compared to the control group. Understanding how task specific information and instruction can impact peak performance in people with CMSK pain requires further investigation.

## Supporting information

S1 Table Explicit motor learning: Within group and between group differences in hand path distance. (DOCX)

S2 Table Total number of errors committed during explicit motor learning. (DOCX)

S3 Table Implicit motor learning: Within group and between group differences in hand path distance. (DOCX)

S4 Table Total number of errors committed during implicit motor learning. (DOCX)

S1 File CNP demographic survey. (DOCX)

S1 Data (XLSX)
