# Supplementary material for: Participants with mildly-disabling chronic neck pain perform differently during explicit compared to implicit motor learning of a reaching task
Source: PLoS One. 2022 Apr 7;17(4):e0266508. doi: 10.1371/journal.pone.0266508 (PMC8989223; doi:10.1371/journal.pone.0266508)
Supplement: S1 Table — (DOCX) [file pone.0266508.s001.docx]

| **S1 Table. Explicit motor learning: within group and between group differences in hand path distance** | | | | | | | | |
| --- | --- | --- | --- | --- | --- | --- | --- | --- |
|  | **Control: Reaching outward** | | | **CNP: Reaching outward** | | | Mann-Whitney | |
| *Block* | *M (cm)* | *SD* | *^a^p-value* | *M (cm)* | *SD* | *^a^p-value* | *U* | *^e^p-value* |
| EB1 | 132.61 | 32.26 |  | 132.95 | 14.98 |  | 136 | 0.419 |
| EB2 | 127.59 | 31.47 | ^b^ 1.000 | 129.28 | 15.29 | ^b^ 1.000 | 143 | 0.558 |
| PRB3* | 214.56 | 65.16 | ^c^ <0.001 | 205.61 | 35.77 | ^c^ <0.001 | 133 | 0.914 |
| EB16 | 132.92 | 30.01 | ^d^ 0.648 | 134.96 | 20.56 | ^d^ 0.332 | 141 | 0.516 |
|  | **Control: Reaching inward** | | | **CNP: Reaching inward** | | | Mann-Whitney | |
| *Block* | *M (cm)* | *SD* | *^a^p-value* | *M (cm)* | *SD* | *^a^p-value* | *U* | *^e^p-value* |
| EB1 | 120.70 | 29.70 |  | 122.51 | 16.46 |  | 141 | 0.516 |
| EB2 | 120.51 | 29.46 | ^b^ 0.359 | 115.60 | 10.87 | ^b^ 0.332 | 158 | 0.912 |
| PRB3* | 124.99 | 30.62 | ^c^ 0.629 | 117.20 | 15.95 | ^c^ 0.454 | 120 | 0.564 |
| EB16 | 122.54 | 26.12 | ^d^ 1.000 | 116.10 | 15.85 | ^d^ 1.000 | 138 | 0.456 |
| Control n = 19, CNP n = 17  *Control n = 17, CNP n = 16  ^a^ p-values calculated using a Sign test.  ^b^ = EB2 – EB1 (comparison of performance change over explicit motor learning blocks)  ^c^ = PRB3 – EB2 (comparison of pseudo-random catch block to explicit motor learning block)  ^d^ = EB16 – EB2 (comparison of explicit motor learning over time)  U = Mann-Whitney U test statistic, ^e^p-values calculated using a Mann-Whitney U test. | | | | | | | | |
