# Supplementary material for: Participants with mildly-disabling chronic neck pain perform differently during explicit compared to implicit motor learning of a reaching task
Source: PLoS One. 2022 Apr 7;17(4):e0266508. doi: 10.1371/journal.pone.0266508 (PMC8989223; doi:10.1371/journal.pone.0266508)
Supplement: S2 Table — (DOCX) [file pone.0266508.s002.docx]

| **S2 Table. Total number of errors committed during explicit motor learning** | | | | | | | | |
| --- | --- | --- | --- | --- | --- | --- | --- | --- |
|  | **Control** | | | **CNP** | | | Mann-Whitney | |
| *Block* | *Sum* | *Range* | *^a^p-value* | *Sum* | *Range* | *^a^p-value* | *U* | *^e^p-value* |
| EB1 | 38 | 6 |  | 34 | 10 |  | 168 | 0.747 |
| EB2 | 35 | 9 | ^b^ 1.000 | 25 | 5 | ^b^ 0.727 | 158.5 | 0.542 |
| PRB3 | 18 | 9 | ^c^ 0.006 | 15 | 4 | ^c^ 1.000 | 156 | 0.458 |
| EB16 | 57 | 9 | ^d^ 0.143 | 39 | 6 | ^d^ 0.070 | 169 | 0.777 |
| Control n = 21, CNP n = 17  Sum = the total number of errors committed by the group  ^a^ p-values calculated using a Sign test.  ^b^ = EB2 – EB1 (comparison of errors between explicit motor learning blocks)  ^c^ = PRB3 – EB2 (comparison of errors pseudo-random catch block to explicit motor learning block)  ^d^ = EB16 – EB2 (comparison of errors over time)  U = Mann-Whitney U test statistic, ^e^p-values calculated using a Mann-Whitney U test. | | | | | | | | |
