# Supplementary material for: Participants with mildly-disabling chronic neck pain perform differently during explicit compared to implicit motor learning of a reaching task
Source: PLoS One. 2022 Apr 7;17(4):e0266508. doi: 10.1371/journal.pone.0266508 (PMC8989223; doi:10.1371/journal.pone.0266508)
Supplement: S3 Table — (DOCX) [file pone.0266508.s003.docx]

| **S3 Table. Implicit motor learning: within group and between group differences in hand path distance** | | | | | | | | |
| --- | --- | --- | --- | --- | --- | --- | --- | --- |
|  | **Control: Reaching outward** | | | **CNP: Reaching outward** | | | Mann-Whitney | |
| *Block* | *M (cm)* | *SD* | *^a^p-value* | *M (cm)* | *SD* | *^a^p-value* | *U* | *^f^p-value* |
| IB5* | 212.56 | 53.31 |  | 197.80 | 30.71 |  | 116 | 0.471 |
| IB8* | 196.83 | 52.92 | ^b^ 0.013 | 191.74 | 39.84 | ^b^ 0.077 | 130 | 0.829 |
| PRB9* | 229.36 | 73.55 | ^c^ 0.002 | 211.97 | 33.66 | ^c^ 0.077 | 120 | 0.564 |
| IB11* | 202.98 | 69.97 | ^d^ 0.629 | 195.32 | 41.37 | ^d^ 0.210 | 126 | 0.801 |
| IB12* | 204.28 | 76.02 |  | 193.17 | 41.60 |  | 129 | 0.801 |
| IB13** | 187.76 | 28.91 | ^e^ 0.454 | 191.75 | 36.67 | ^e^ 1.000 | 122 | 0.821 |
|  | **Control: Reaching inward** | | | **CNP: Reaching inward** | | | Mann-Whitney | |
| *Block* | *M (cm)* | *SD* | *^a^p-value* | *M (cm)* | *SD* | *^a^p-value* | *U* | *^f^p-value* |
| IB5* | 115.42 | 28.09 |  | 108.79 | 8.10 |  | 126 | 0.719 |
| IB8* | 115.35 | 34.37 | ^b^ 0.629 | 112.17 | 13.15 | ^b^ 0.804 | 121 | 0.589 |
| PRB9* | 112.10 | 31.31 | ^c^ 0.143 | 110.03 | 13.12 | ^c^ 0.804 | 115 | 0.449 |
| IB11* | 116.86 | 35.36 | ^d^ 0.629 | 105.03 | 9.70 | ^d^ 0.077 | 106 | 0.280 |
| IB12* | 116.95 | 37.08 |  | 105.08 | 10.56 |  | 118 | 0.517 |
| IB13** | 108.68 | 11.28 | ^e^ 1.000 | 110.30 | 8.66 | ^e^ 0.077 | 109 | 0.474 |
| *Control n = 17, CNP n = 16  **Control n = 16, CNP n = 16  ^a^ p-values calculated using a Sign test  ^b^ = IB8 – IB5 (comparison of performance change over implicit motor learning blocks)  ^c^ = PRB9 – IB8 (comparison of pseudo-random catch block to implicit motor learning block)  ^d^ = IB11 – IB8 (implicit motor learning following disruption)  ^e^ = IB13 – IB12 (implicit motor learning following a 30-minute delay)  U = Mann-Whitney U test statistic, ^f^ p-values calculated using a Mann-Whitney U test | | | | | | | | |
