# Supplementary material for: Participants with mildly-disabling chronic neck pain perform differently during explicit compared to implicit motor learning of a reaching task
Source: PLoS One. 2022 Apr 7;17(4):e0266508. doi: 10.1371/journal.pone.0266508 (PMC8989223; doi:10.1371/journal.pone.0266508)
Supplement: S4 Table — (DOCX) [file pone.0266508.s004.docx]

| **S4 Table. Total number of errors committed during implicit motor learning** | | | | | | | | |
| --- | --- | --- | --- | --- | --- | --- | --- | --- |
|  | **Control** | | | **CNP** | | | Mann-Whitney | |
| *Block* | *Sum* | *Range* | *^a^p-value* | *Sum* | *Range* | *^a^p-value* | *U* | *^f^p-value* |
| IB5 | 13 | 3 |  | 9 | 2 |  | 140 | 0.656 |
| IB8 | 19 | 4 | ^b^ 0.791 | 7 | 3 | ^b^ 1.000 | 94.5 | 0.036 |
| PRB9 | 12 | 2 | ^c^ 0.388 | 10 | 2 | ^c^ 0.344 | 151 | 0.971 |
| IB11 | 15 | 3 | ^d^ 0.581 | 7 | 1 | ^d^ 0.727 | 124.5 | 0.312 |
| IB12 | 17 | 5 |  | 8 | 3 |  | 126 | 0.327 |
| IB13 | 20 | 4 | ^e^ 0.549 | 9 | 3 | ^e^ 1.000 | 112 | 0.150 |
| Control n = 21, CNP n = 17  Sum = the total number of errors committed by the group  ^a^ p-values calculated using a Sign test  ^b^ = IB8 – IB5 (comparison of performance change over implicit motor learning blocks)  ^c^ = PRB9 – IB8 (comparison of pseudo-random catch block to implicit motor learning block)  ^d^ = IB11 – IB8 (comparison of errors following disruption)  ^e^ = IB13 – IB12 (comparison of errors following 30-minute break)  U = Mann-Whitney U test statistic, ^f^ p-values calculated using a Mann-Whitney U test | | | | | | | | |
