# Supplementary material for: Participants with mildly-disabling chronic neck pain perform differently during explicit compared to implicit motor learning of a reaching task
Source: PLoS One. 2022 Apr 7;17(4):e0266508. doi: 10.1371/journal.pone.0266508 (PMC8989223; doi:10.1371/journal.pone.0266508)

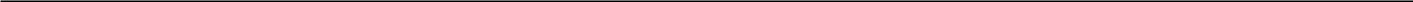


1. **How long has neck pain been an ongoing problem for you?**
   - Less than 1 month
   - 1–3 months
   - 3–6 months
   - 6 months–1 year
   - 1–5 years
   - More than 5 years
2. **How often has neck pain been an ongoing problem for you over the past 6 months?**
   - Every day or nearly every day in the past 6 months
   - At least half the days in the past 6 months
   - Less than half the days in the past 6 months
3. **In the past 7 days, how would you rate your neck pain on average?**

|  |  |  |  |  |  |  |  |  |  |
| --- | --- | --- | --- | --- | --- | --- | --- | --- | --- |
| 1 | 2 | 3 | 4 | 5 | 6 | 7 | 8 | 9 | 10 |
| No pain |  |  |  |  |  |  |  |  | Worst |
|  |  |  |  |  |  |  |  |  | Imaginable |
|  |  |  |  |  |  |  |  |  | pain |

1. **Has neck pain spread down your arm(s) during the past 2 weeks?**
   - Yes
   - No
   - Not sure
2. **During the past 4 weeks, how much have you been bothered by …**

x Stomach pain

- Pain in your arms, legs, or joints other than your spine or back
- Headaches
- Widespread pain or pain in most of your body

| **Not bothered** | **Bothered a little** | **Bothered a lot** |
| --- | --- | --- |
| **at all** |  |  |
|  |  |  |
|  |  |  |
|  |  |  |
|  |  |  |
|  |  |  |

1. **Have you ever had a neck operation?**
   - Yes, one operation
   - Yes, more than one operation
   - No


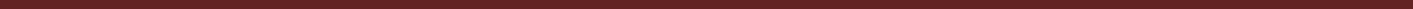

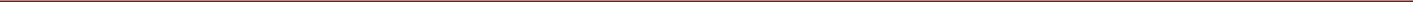


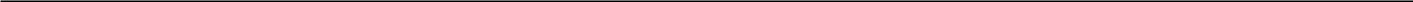


1. **If yes, when was your last neck operation?**
   - Less than 6 months ago
   - More than 6 months but less than 1 year ago
   - Between 1 and 2 years ago
   - More than 2 years ago
2. **Did any of your neck operations involve a spinal fusion? (also called an arthrodesis)**
   - Yes
   - No
   - Not sure

| **In the past 7 days…** | | **Not at all** | **A little** | **Somewhat** | **Quite a** | **Very** |
| --- | --- | --- | --- | --- | --- | --- |
| **9.** | **How much did pain interfere with** |  | **bit** |  | **bit** | **much** |
|  |  |  |  |  |  |  |
|  | **your day-to-day activities?** |  |  |  |  |  |
| **10.** | **How much did pain interfere with** |  |  |  |  |  |
|  | **work around the home?** |  |  |  |  |  |
| **11.** | **How much did pain interfere with** |  |  |  |  |  |
|  | **your ability to participate in social** |  |  |  |  |  |
|  | **activities?** |  |  |  |  |  |
| **12.** | **How much did pain interfere with** |  |  |  |  |  |
|  | **your household chores?** |  |  |  |  |  |

**13. Have you used any of the following treatments for your neck pain? *(Check all that apply)***

**Not**

**Yes** **No**

| x **Opioid painkillers** (*prescription medications such as Vicodin, Lortab,* |  |  | **sure** |
| --- | --- | --- | --- |
|  |  |  |  |
| *Norco, hydrocodone, codeine, Tylenol #3 or #4, Fentanyl, Duragesic, MS* |  |  |  |
| *Contin, Percocet, Tylox, OxyContin, oxycodone, methadone, tramadol,* |  |  |  |
|  |  |  |  |
| *Ultram, Dilaudid*) |  |  |  |
| **If you checked yes, are you currently using this medication?………….** |  |  |  |
| x **Injections** *(such as epidural steroid injections, facet injections)* **……………..** |  |  |  |
| x **Exercise therapy…………………………………………………………………………………..** |  |  |  |
| x **Psychological counseling, such as cognitive-behavioral** **therapy……………** |  |  |  |
|  |  |  |  |
| **The next two questions are for people who normally work outside the home.** |  |  |  |

1. **I have been off work or unemployed for 1 month or more** due to neck pain.
   - Agree
   - Disagree
   - Does not apply


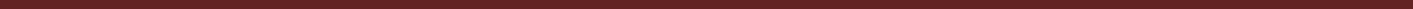

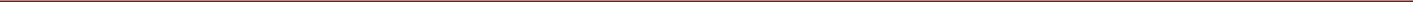


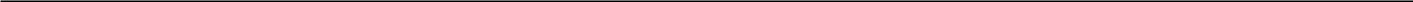


1. **I receive or have applied for disability or workers’ compensation benefits because I am unable to work due to neck pain.**
   - Agree
   - Disagree
   - Does not apply

**Physical Function**

1. **Are you able to do chores such as vacuuming or yard work?**
2. **Are you able to lift and carry groceries?**
3. **Are you able to participate in recreational activities or hobbies?**
4. **Are you able to drive your car community distances?**

**In the past 7 days...**

1. **I felt worthless**
2. **I felt helpless**
3. **I felt depressed**
4. **I felt hopeless**

**In the past 7 days…**

1. **My sleep quality was**

**In the past 7 days…**

1. **My sleep was refreshing**
2. **I had a problem with my sleep**
3. **I had difficulty falling asleep**

| **Without** | **With a** | **With** | **With** | **Unable** |
| --- | --- | --- | --- | --- |
| **any** | **little difficulty** | **some** | **much difficulty** | **to do** |
| **difficulty** |  | **difficulty** |  |  |
|  |  |  |  |  |
|  |  |  |  |  |
|  |  |  |  |  |
|  |  |  |  |  |
| **Never** | **Rarely** | **Sometimes** | **Often** | **Always** |
|  |  |  |  |  |
|  |  |  |  |  |
|  |  |  |  |  |
|  |  |  |  |  |
| **Very poor** | **Poor** | **Fair** | **Good** | **Very good** |
|  |  |  |  |  |
| **Not at all** | **A little bit** | **Somewhat** | **Quite a bit** | **Very much** |
|  |  |  |  |  |
|  |  |  |  |  |
|  |  |  |  |  |

**
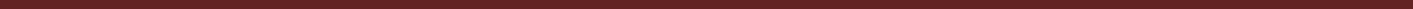

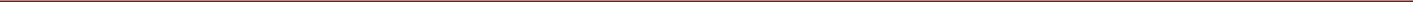
**


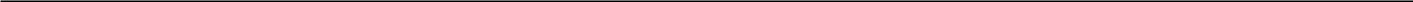


1. **It’s not really safe for a person with my neck problem to be physically active.**
   - Agree
   - Disagree
2. **I feel that *my neck pain is terrible* and *it’s never going to get any better*.**
   - Agree
   - Disagree
3. **Are you involved in a lawsuit or legal claim related to your neck problem?**
   - Yes
   - No
   - Not sure

| **In the past year:** | **Never** | **Rarely** | **Sometimes** | **Often** |
| --- | --- | --- | --- | --- |
| **31. Have you drunk or used drugs more than you** |  |  |  |  |
|  |  |  |  |  |
| **meant to?** |  |  |  |  |
| **32. Have you felt you wanted or needed to cut** |  |  |  |  |
| **down on your drinking or drug use?** |  |  |  |  |
|  |  |  |  |  |

1. **Age: _____** years (0–120)
2. **Gender:**
   - Female
   - Male
   - Unknown
   - Unspecified
3. **Ethnicity:** *(“X” ONLY one with which you MOST CLOSELY identify)*
   - Hispanic or Latino
   - Not Hispanic or Latino
   - Unknown
   - Not Reported
4. **Race:** *(“X” those with which you identify)*
   - American Indian or Alaska Native
   - Asian
   - Black or African-American
   - Native Hawaiian or Other Pacific Islander
   - White
   - Unknown
   - Not Reported


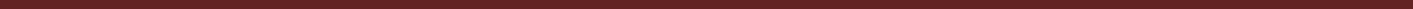

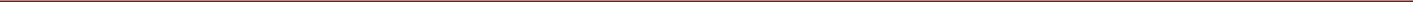


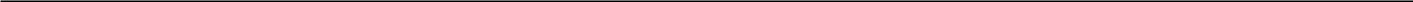


1. **Employment Status:**
   - Working now
   - Looking for work, unemployed
   - Sick leave or maternity leave
   - Disabled due to back pain, permanently or temporarily
   - Disabled for reasons other than back pain
   - Student
   - Temporarily laid off
   - Retired
   - Keeping house
   - Other, Specify:_________________
   - Unknown
2. **Education Level:** *(select the highest level attained)*
   - No high school diploma
   - High school graduate or GED
   - Some college, no degree
   - Occupational/technical/vocational program
   - Associate degree: academic program
   - Bachelor’s degree
   - Master’s degree (e.g., M.A., M.S., M.Eng., M.Ed., M.B.A.)
   - Professional school degree (e.g., M.D., D.D.S., D.V.M., J.D.)
   - Doctoral degree (e.g., Ph.D., Ed.D.)
   - Unknown
3. **How would you describe your cigarette smoking?**
   - Never smoked
   - Current smoker
   - Used to smoke, but have now quit

**40. Height:** _____  inches  centimeters  measured  self-reported

**Weight: ____**  pounds  kilograms  measured  self-reported


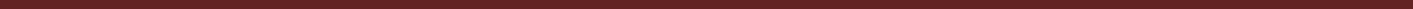

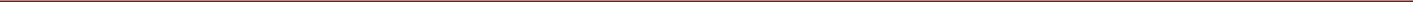

Supplement: S1 File — (DOCX) [file pone.0266508.s005.docx]
